# Biological Prosthesis (Hollow 3D-Printed Titanium Custom-Made Prosthesis and Bone Graft) for Humeral Reconstruction in Pediatric Oncologic Patients: Surgical Indications and Results

**DOI:** 10.3390/bioengineering10121371

**Published:** 2023-11-29

**Authors:** Giovanni Beltrami, Sreeraj Rajan, Anna Maria Nucci, Alberto Galeotti, Davide Guido, Domenico Campanacci, Marco Innocenti

**Affiliations:** 1 Department of Orthopedic, Traumatology and Paediatric Orthopaedic Oncology, Azienda Ospedaliero Universitaria IRCCS, Meyer Children Hospital, 50139 Florence, Italy; 2 Department of Orthopaedic Oncology, Aster MIMS, Calicut 673016, India; 3 Department of Orthopedic Oncology and Reconstructive Microsurgery, CTO, 10100 Florence, Italy; 4 Orthoplastic Surgery Division, Istituto Ortopedico Rizzoli, 40136 Bologna, Italy

**Keywords:** bone sarcoma, pediatric limb salvage, biological prosthesis, 3D-printed custom-made prosthesis, articular bone sparing

## Abstract

This study presents the mid-term outcomes of a novel “biological prosthesis” for pediatric humerus reconstruction after major bone tumor removal. This approach involves a hollow 3D-printed titanium custom-made prosthesis combined with bone grafting. The primary aim was to preserve and revitalize the unaffected autologous proximal or distal humeral stump. Between 2017 and 2021, we treated five pediatric patients (mean age 11.2 years; range 7–17) with humeral bone sarcomas. A one-stage surgical procedure involved tumor resection and implanting a hollow 3D-printed custom-made prosthesis. In two cases, we preserved the proximal humerus; in two, the distal part; and in one, both. Graft materials included homologous bone chips in three cases and free vascularized fibular grafts in two cases. All patients were clinically and radiographically assessed after a mean follow-up of 32.2 months (range of 14–68). No significant complications were observed, and no implant revisions were needed. Osseointegration was evident in all cases within eight months post-surgery; vascular support for the remaining autologous stump was demonstrated in all cases. Our hollow 3D-printed custom-made prosthesis and bone grafting offer the potential for partial or complete articular surface preservation. This approach encourages revascularization of the epiphysis, leading to satisfactory outcomes in humerus reconstruction within the pediatric population.

## 1. Introduction

Bone sarcomas are relatively rare in children, accounting for 8% of all pediatric cancers [1]; the most common sites are the femur, tibia, and humerus [2]. Advances in imaging, chemotherapy, bioengineering, and surgical techniques have made limb salvage surgery the standard of care for most patients with bone sarcomas, providing better functional outcomes without compromising oncological outcomes [3]. Achieving adequate margins during wide local excision ensures the surgery’s local and overall outcomes [4], often leaving large and complex bone and soft tissue defects. Reconstruction of the remaining bone defects is a challenge for orthopedic oncology surgeons.

Limb reconstruction aims to restore the upper limb’s morphology and function. Restoration is essential for children who need their upper limbs for daily living, playing, and overall quality of life. Pediatric patients also have specific needs in terms of limb reconstruction. For example, the dimensions of standard prostheses may not be compatible with the smaller bones of children, and the reconstruction must consider the child’s residual growth potential. Moreover, reconstruction must have solid primary and effective secondary stability due to the high functional and biomechanical demands in these patients.

Over time, various surgical techniques have been used to reconstruct significant bone defects in children after a wide resection for humeral bone sarcoma. These techniques include biological reconstruction with bone allografts, bone autografts (vascularized fibula or clavicle pro humerus), or free vascularized bone grafts, and extracorporeal irradiated tumor bone have been widely used [5,6,7,8,9].

Effective long-term outcomes are associated with these techniques, but they have drawbacks. Despite providing immediate structural support and the potential for ligament and tendon reattachment, lengthy surgeries and extensive exposure increase infection risk, osteotomy nonunion, wound-healing issues, and donor site morbidity (in autograft cases). Additionally, graft molding for defect fitting is technically challenging and time-consuming [5,8].

Prosthetic reconstruction (e.g., conventional, composite, expandable prosthesis, and mega-prosthesis) offers stable primary fixation and rapid recovery. However, it comes with common complications (aseptic loosening, infection, and periprosthetic fracture) [10,11,12].

Advancements in bioengineering and computer-aided design (CAD) enable 3D-printed custom prostheses tailored to individual digital plans. Custom prostheses offer numerous advantages. They utilize 3D-printed cutting guides for precise pre-planned osteotomies, speeding up surgery and reducing blood loss and infection risk. Hollow 3D-printed custom prostheses, coated with bone graft-filled porous material, have been proposed for oncological resections to enhance secondary fixation [13]. Furthermore, this technique rejuvenates proximal or distal residual autologous periarticular stumps, improving function without affecting contralateral articular surfaces and the physis. This paper presents the surgical technique and outcome of a consecutive series of five humeral reconstructions treated using a biological prosthesis (a hollow 3D-printed titanium custom-made prosthesis and bone graft) in pediatric oncological patients.

## 2. Materials and Methods

From September 2017 to March 2021, five female pediatric patients with primary malignant humerus bone tumors underwent salvage surgery using hollow, custom-made, 3D-printed implants. This retrospective study examined this patient series (Table 1). The mean age at surgery was 11.2 years (range 7–17), with histology revealing Ewing’s sarcoma in four patients and one case of osteosarcoma. Oncological staging classified four patients as having localized disease, while one presented with lung metastasis. Three patients had undisplaced pathological fractures initially, which healed conservatively during neoadjuvant chemotherapy, and none received radiotherapy.

All patients underwent preoperative evaluations to identify distant metastases and tumor extent. The evaluations included conventional radiographs, computerized axial tomography, magnetic resonance imaging, core needle biopsy for histological diagnosis, and whole-body positron emission tomography. Tumors were classified and staged based on the Tumor, Node, Metastasis (TNM) staging system [14]. These cases were discussed in multidisciplinary tumor board meetings, and all patients received neoadjuvant chemotherapy with OS2 PGP-based regimens for osteosarcoma and ISG/AIEOP EW-1/2 for Ewing’s sarcoma.

The surgical procedure and custom implant manufacturing began once the patient’s parents provided written informed consent. Initially, a CAD model of the affected bone segment was created using computed tomography (CT) scan data. The surgeon determined the location of the osteotomies, and the custom implant was designed to fit the gap after bone resection. The osteotomy design allowed for preserving at least one epiphysis (proximal or distal). The implant design also included specifications for securing the device to the host bone, such as screws, stems, plates, and modular components.

Reconstruction involved using a titanium custom-made implant, including total humerus hollow custom-made prostheses with proximal epiphysis preservation (n = 2), a total humerus hollow custom-made prosthesis with preservation of both proximal and distal epiphyses (n = 1), a proximal and subtotal humerus hollow custom-made prosthesis connected to an anatomical plate on the distal stump (n = 1), and an extra-articular total shoulder resection with a conventional glenoid component and a proximal humerus hollow custom-made prosthesis connected to an anatomical plate on the distal stump (n = 1).

**Figure 1:**
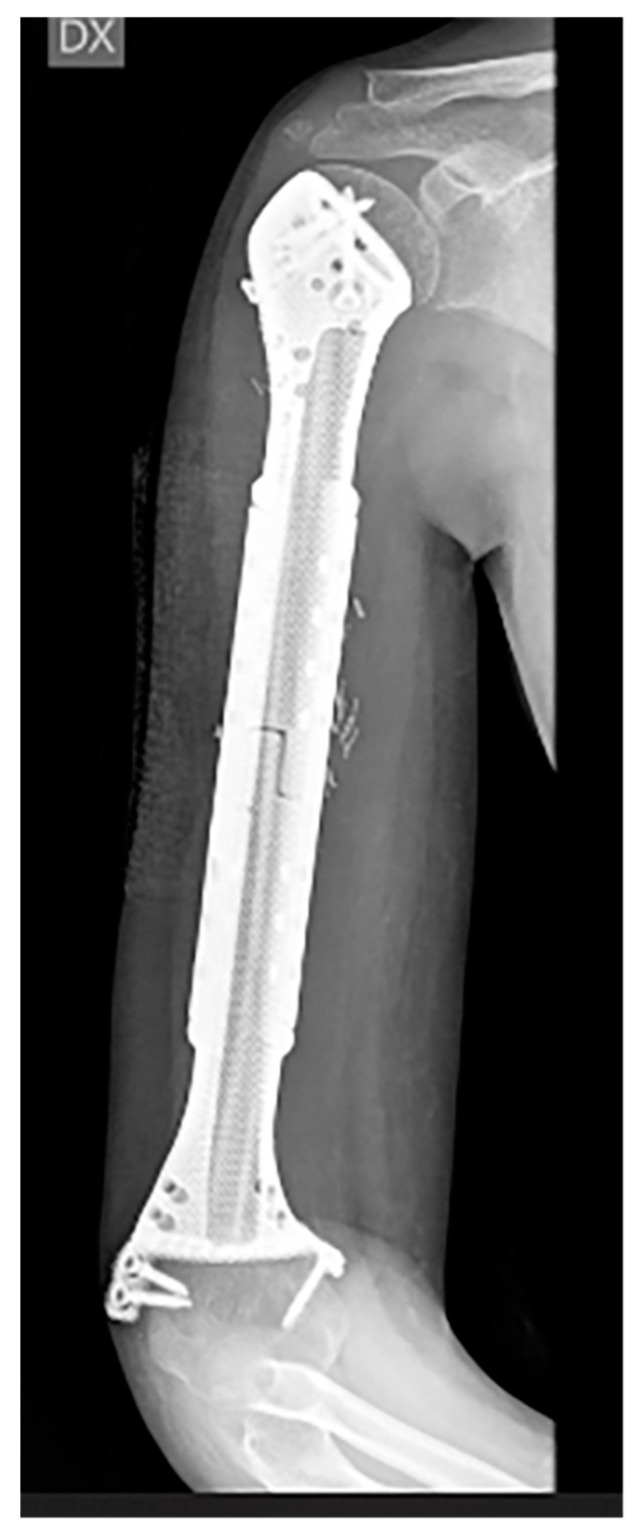
A 10-year-old child with Ewing’s sarcoma presented with subtotal humeral involvement. The patient underwent a proximal trans-epiphyseal resection and reconstruction using a custom-made 3D hollow prosthesis containing a vascularized fibula graft while preserving the proximal epiphysis and distal metaphyseal epiphysis.

**Figure 2:**
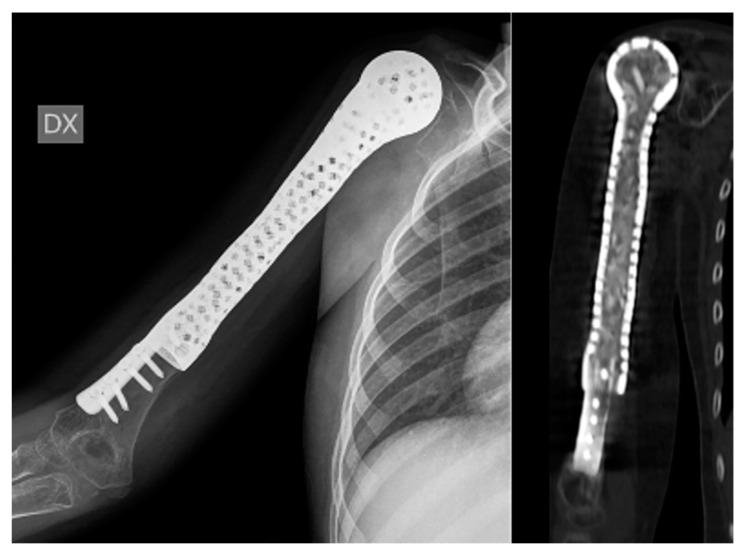
A 7-year-old child diagnosed with Ewing’s sarcoma with proximal humeral involvement underwent resection and reconstruction utilizing a 3D custom-made hollow prosthesis containing allograft cortico-cancellous bone chips while preserving the distal humerus.

These hollow prostheses featured one or more grooves designed for bone graft placement to enhance integration with the remaining host bone. Emphasis was placed on bone graft augmentation, with two patients receiving vascularized fibular grafts performed by a microsurgical team and three receiving allograft corticocancellous bone graft chips. All implants were covered by vital autologous muscles, which facilitated the vascularization of allograft chips through multiple perforations in the custom-made prosthesis. The choice of graft depended on the resection length and peri-articular stump size. For proximal trans-epiphyseal resections, we vascularized the residual epiphysis with a vascularized fibula graft, while for meta-epiphyseal residual stumps, conventional morselized bone grafts were used, revitalized through custom holes (see Figure 1 and Figure 2).

**Figure 3:**
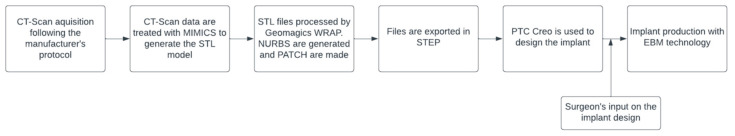
Custom implant design and fabrication process.

Custom implants were created in collaboration with an industry partner (Adler Orthopedics S.p.A., Cormano, Milano, Italy), following a specific procedure (Figure 3). First, Computed Tomography (CT) scans of the affected anatomical segment were conducted following the scanning protocol provided by the implant manufacturer, with a scan thickness of 0.6 mm. It is worth noting that Magnetic Resonance Imaging (MRI) was exclusively used to assess the extent of the area to be resected, not for anatomical reconstruction or implant design.

The CT scan data were then processed using Mimics to extract the Stereolithography (STL) model of the anatomical segment. Subsequently, the file was imported into Geomagics WRAP (3D Systems, Rock Hill, SC, USA), where any potential artifacts and disturbances were meticulously removed. In some cases, minor smoothing was applied to selected areas. The same software was employed to generate Non-Uniform Rational B-Splines (NURBS), utilizing an algorithm capable of creating PAtCHes on anatomical structures and exporting them in Standard for the Exchange of Product Data (STEP) format. The resulting data were leveraged in the design of both the implant and the associated instruments using the software PTC Creo (PTC, Boston, MA, USA). The implant design closely adhered to the surgeon’s specifications, accommodating necessary space for vascularized bone grafts and other requirements.

With regard to the design of the implant, the process began with a comprehensive risk analysis, where specific areas of potential risk were identified. Subsequently, the manufacturer conducted Finite Element analysis, focusing on the critical aspects of the implant.

Once the implant design received validation from the surgeon, the production phase commenced, employing Electron Beam Melting (EBM) technology. EBM is a cutting-edge technique for sintering titanium powder, enabling the production of implants that match the 3D reconstruction precisely. This process utilizes a high-energy electron beam to melt titanium powder within a vacuum chamber. Disposable patient-specific instruments and trial components were typically three-dimensionally (3D) printed using medical-grade nylon. Manufacturing time averaged approximately 4–5 weeks in all cases.

Following neoadjuvant chemotherapy, preoperative local Magnetic Resonance Imaging (MRI) and CT lung scans were conducted. Subsequently, the patients underwent bone tumor resection and biological prosthesis reconstruction. Surgery duration varied primarily based on the type of reconstruction, with conventional allograft cases averaging five hours and vascularized fibula transplants averaging nine hours.

After surgery, patients with proximal humerus-sparing cases had their limbs placed in a 90-degree sling, while those with distal humerus-sparing cases used conventional slings. Orthotics were provided for two months post-surgery, followed by physiotherapy to aid functional recovery.

All patients were referred to the pediatric oncology department for ongoing medical adjuvant treatment and subsequent oncological follow-up. Complications were categorized using the Henderson classification [15], encompassing soft tissue failures (musculo-ligamentous deficiencies or wound dehiscence), aseptic loosening, structural failures (implant breakage, graft fractures, or peri-prosthetic fractures), infections, and tumor progression. Graft-host nonunion was also considered a potential complication. Pediatric-specific issues, such as physeal arrest or dysplastic joints resulting from implant articulation, were also considered. Functional outcomes were assessed using the MSTS score for the upper limb [16].

## 3. Results

The mean follow-up duration was 32.2 months (range: 14–68). All patients initially underwent surgery for tumor resection, with reported wide margins based on intraoperative histological analysis. No major complications were documented. Specifically, no superficial or deep infections were observed, and there were no mechanical issues requiring treatment. One patient reported occasional mild pain attributed to soft tissue impingement with the head of a screw, which did not necessitate surgical revision.

Partial osseointegration between various grafts and host bone was radiographically observed within a mean of 4 months (range: 3–6), while complete osseointegration typically occurred within a mean of 7 months (range: 6–8). Notably, there were no significant differences in healing time or osteotomy fusion between the vascularized fibular graft and bone allograft chips groups. No necrosis or deformity of the residual stump was observed at the final follow-up. In the two cases involving prosthetic proximal humerus surfaces, the functional results were poorer compared to the three cases of proximal humerus epiphyseal sparing.

The range of motion for the two cases of distal osteoarticular prosthetic implants was rated as satisfactory, with full flexion and pronation–supination. However, a 50° limitation of extension was noted, which did not hinder daily activities. This limitation in extension was anticipated and addressed with a synthetic band to minimize and delay potential osteoarticular wear due to the differing modulus of elasticity between the polished titanium surface and cartilage. Both patients reported personal satisfaction with their elbow range of movement and were symptom-free.

Overall, functional outcomes were generally satisfactory (see Table 2), with a mean score based on the MSTS 93 system of 82% (range: 66–93%). At the final follow-up, three patients were alive with no evidence of disease (NED), one was alive with disease (AWD), and unfortunately, one patient passed away due to disease progression 18 months after surgery.

## 4. Discussion

The optimal approach for humerus bone resection after sarcoma surgery remains debatable. Reconstructive options should preserve bone growth potential and long-term limb function. Many authors have proposed various techniques for humeral tumor reconstructions.

Multiple studies support osteoarticular allografts for proximal humerus resection, enabling an anatomical reconstruction of the resected bone segment by preserving the graft’s joint surface. However, these studies also report high revision rates due to resorption, fractures, and infections [8,17]. Depending on the involvement of the rotator cuff, allograft composite prostheses with or without reverse shoulder arthroplasty are alternatives in case of proximal humerus sarcomas, with complications such as delayed union, dislocation, deep infection, graft fracture, delayed wound healing, and periprosthetic fracture [12,18]. Arthrodesis may suit significant volume diseases involving deltoid muscles or axillary nerves but often results in limited function, poor aesthetics, nonunion, and infections [19]. Clavicula pro-humero suspension is useful when sparing the deltoid, not the glenoid and scapular neck [6]. To address limb length discrepancy, techniques include vascularized free fibula epiphyseal transfer (VFET) and expandable prostheses, with VFET having a high failure rate, avascular necrosis, and graft fracture [20,21]. Extendable prostheses report complications in 37% of patients [10], and VFET is mainly indicated until age six, when growth plate transplant and remodeling are more effective.

Diaphyseal lesions use allografts or recycled autografts, often reinforced with intramedullary cement or inlay vascularized/non-vascularized fibular grafts. They offer biological solutions but risk nonunion, infection, and fracture [22,23,24]. Distal humeral reconstruction remains complex due to elbow joint intricacies, limited soft tissue coverage, and proximity to nerves and arteries. High complication and revision rates are reported, particularly for allografts [25,26].

**Figure 4:**
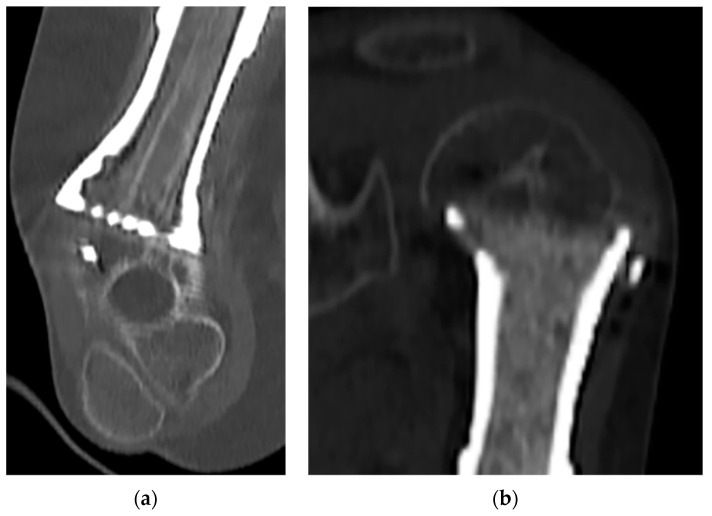
(**a**) Vascularized fibular graft with superior healing capabilities, which faced a significant size discrepancy in terms of healing potential between the humerus epiphysis and the fibular graft size. (**b**) Allograft bone chips, which are less vascularized but share identical contact surfaces with the humerus epiphysis.

In our series, we noted no significant differences in healing time or osteotomy fusion between the vascularized fibular graft and bone allograft chips groups. This can be explained by the fact that the vascularized fibular graft, with its higher healing capability, had a more significant size discrepancy to heal (humerus epiphysis vs. fibular size), while allograft bone chips, being less vascularized, had similar contact surfaces (see Figure 4a,b). Our finding that no necrosis or deformity of the residual stump was observed at the final follow-up confirms the effectiveness of the revascularization process.

Functional outcomes were less favorable in the two instances where prosthetic proximal humerus surfaces were involved, in contrast to the three cases where proximal humerus epiphyses were preserved. This underscores the critical need to spare the proximal native humeral head when oncologically feasible, especially in the pediatric population.

A disparity in upper limb length was noted in four patients. While we acknowledge the significance of preventing disparities in upper limb length, particularly in younger patients, it is crucial to underscore that our approach prioritized upper limb functionality, joint preservation, and the overall patient experience. In cases involving the upper limbs, patients frequently demonstrate a greater tolerance for variations in limb length when compared to the lower limbs.

The recent development of 3D custom-made implants signifies a cutting-edge innovation in limb salvage surgery [13,27,28,29]. These implants offer numerous advantages in size and shape customization tailored to each patient’s defect and functional requirements. Unlike conventional and modular prostheses, 3D implants can precisely match the resected bone’s size, preventing issues like mismatching, oversizing, stress shielding, periprosthetic fractures, and aseptic loosening often associated with adult-sized prosthetics used in pediatric cases. Customized implants also outperform massive allografts, typically harvested from adult donors, by eliminating the need for manual carving to fit pediatric patients’ sizes. Their precise fit results in superior biomechanical stability and strength.

Furthermore, 3D implants can incorporate articular surfaces (either partial or complete), customized plates, or integrated plate prosthesis systems. Coupled with patient-specific jigs, these implants allow precise cuts near the physis, preserving them without compromising margins. Additionally, removable devices, especially anatomical plates, can be integrated to offer temporary mechanical reinforcement, removable when their purpose is fulfilled or for revision purposes. This approach reduces the reliance on expandable prostheses and complex procedures, such as VFET, when feasible. Custom-made 3D implants also permit optional insertion sites for tendons, ligaments, or capsules, enhancing primary fixation through planned preoperative screw placement. Long-term implant survival hinges on secondary stability and is facilitated by integrating the inlay bone prosthesis and native bone.

Beltrami et al. have proposed innovative hollow 3D-printed custom-made prostheses with porous coatings filled with bone grafts for oncological resections, combining the benefits of 3D custom-made prostheses with the biological properties of bone grafts and microsurgical flaps [30,31]. This technique is groundbreaking, as it can potentially combine the advantages of 3D-printed custom-made prostheses with the biological properties of bone grafts and microsurgical flaps. It is hypothesized that the combination of the porous coating and the presence of the bone graft within the prosthesis provides a better scaffold for osteo-induction and osteo-conduction, leading to increased osteo-integration and bone growth. This, in turn, ensures stable secondary fixation, making the implant resistant to torsional and bending forces and reducing long-term mechanical complications. Additionally, when epiphysis-sparing bone resections are performed, the possibility of filling these hollow prostheses with vascularized grafts or revascularized bone chips allows for their vascularization and early union. These prostheses are versatile and suitable for joint reconstructions while respecting articular surface anatomy or reconstructing bone segments with complex anatomy. Since their introduction in 2016, this technique has shown reproducibility and effectiveness, particularly in pediatric cases, enabling a broader range of shoulder joint motion when preserving the revascularized residual physis. Overall, intercalary or osteo-articular “biological prosthesis” implants have proven to be solid and definitive, combining the mechanical strength of titanium with the elasticity and healing capacity of revascularized bone. Furthermore, “sparing the more native bone when possible” without compromising surgical margins allows for normal growth of the contralateral articular surface. This is a benefit not typically achievable with conventional prostheses.

**Figure 5:**
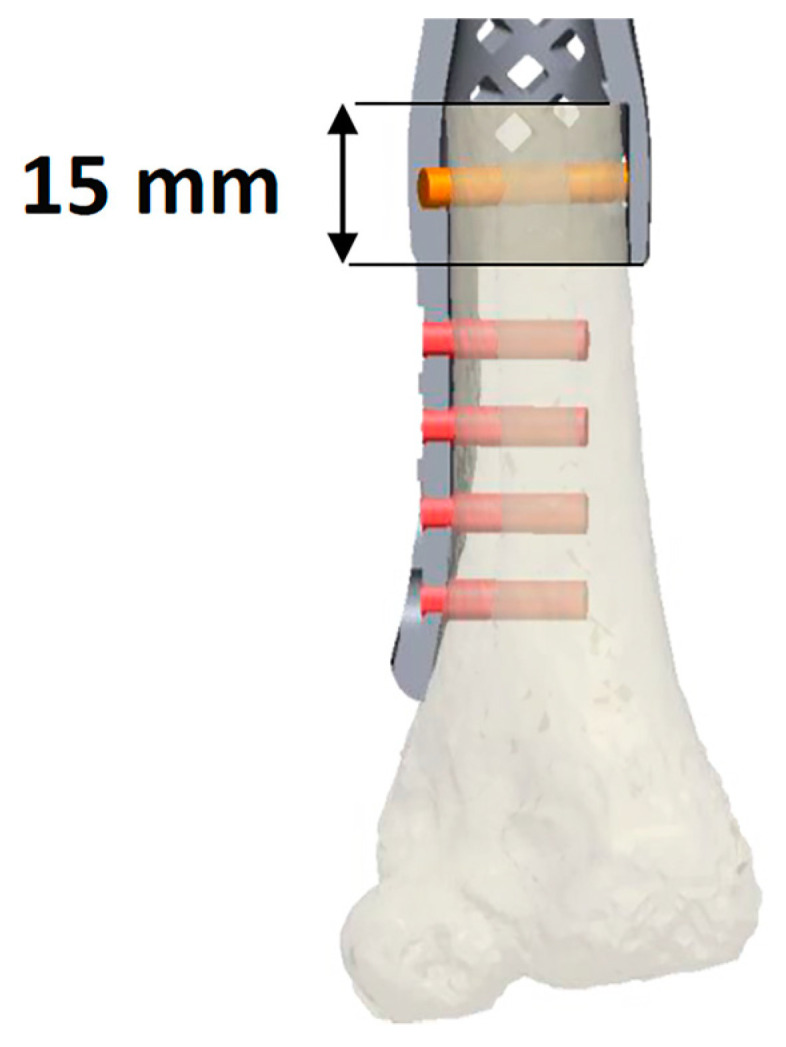
This figure depicts osteotomy of the residual native bone through the wider end of the prosthesis to allow circumferential accommodation of the distal bone. This enables the bone to fit securely into the prosthesis, thereby enhancing overall stability.

In delineating the uniqueness of this construct within the medical realm, we have honed in on specific technical intricacies. We underscored the significance of a wider prosthesis end to accommodate the distal bone circumferentially (Figure 5). This adaptation fosters superior osseointegration and bolsters stability, concurrently mitigating the typical torsional and distraction forces that lead to the loosening of conventional endo-medullary prosthetic stems. Moreover, we advocate preserving the native epiphysis whenever oncologically feasible, be it proximal or distal, even in cases where the residual articular segment is small. Revascularization through the judicious use of bone grafts is a pivotal factor in enhancing functional outcomes.

Furthermore, we endorse the implementation of hollow titanium custom-made implants, especially when designated for VFGs or implants with multiple superficial holes for revascularizing bone allograft chips. This approach, referred to as the “biological prosthesis”, has emerged as exceptionally effective, boasting both durability and robust osteointegration.

**Figure 6:**
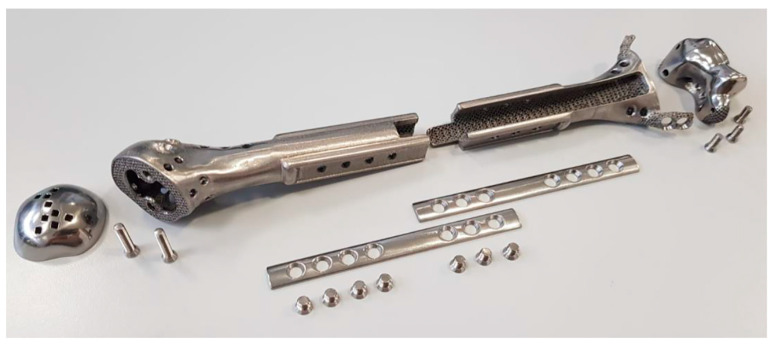
This figure illustrates the modularity of the designed prosthesis, featuring detachable components.

Finally, considering pediatric patients’ potential need for total or partial implant revision, we advocate for comprehensive modularity within the implant design. This modularity permits the revision of individual elements as needed, eliminating the necessity for wholesale replacement in instances of partial failure, as delineated in Figure 6.

This study has several limitations. First, its retrospective nature and limited clinical cases may introduce selection bias. Second, despite achieving favorable outcomes, the mean follow-up period of 32 months might not be sufficiently long to demonstrate long-term effects. Third, compared to a standard prosthesis, the associated high costs, human resources, and time requirements may render the procedure impractical for all, especially in emergencies. Our study primarily focuses on mid-term clinical outcomes and the feasibility of using 3D-printed prostheses for pediatric humerus reconstruction. While we acknowledge the importance of cost considerations in healthcare, it is essential to note that a comprehensive cost-effectiveness assessment requires a longer evaluation, including long-term patient outcomes and technology cost evolution. Given the novelty of this technology, drawing definitive conclusions about its cost-effectiveness in comparison to traditional methods is premature. Early adoption of innovative medical technologies often entails higher initial costs, which may decrease as technology becomes more widespread and efficient. Ongoing research and field advancements will provide a more comprehensive understanding of the financial implications of this technique.

## 5. Conclusions

Overall, hollow 3D-printed custom-made prostheses filled with bone grafts have the potential to combine the advantages of 3D-printed custom-made prostheses with the biological properties of bone grafts and microsurgical flaps. Furthermore, this technique facilitates partial or total articular surface preservation thanks to the bone graft that ensures revascularization of the epiphysis. Moreover, we observed that sparing the epiphysis whenever feasible improved functional outcomes. Our report highlights the satisfactory results achieved with this technique in humerus reconstruction within the pediatric population following wide bone tumor resection, characterized by robust osseointegration and the absence of significant complications. However, further studies involving larger cohorts and longer follow-up periods would confirm and substantiate our findings.

## Figures and Tables

**Table 1:** Baseline characteristics of the study population.

Patient	Sex	Age	Anatomical Site	Histology	TNM Staging	Pathologic Fracture at Diagnosis	Mets at Diagnosis	Concomitant Therapy	Surgical Time (Min)	Quality of the Surgical Margins	Bone/Spacer
1	F	14	Diaphysis	OS	T2 M0 N0	Yes	No	OS2 PGP	600	R0	VFG
2	F	17	Meta-physis prox	Ewing	T2 M0 N0	Yes	No	ISG/AIEOP EW-1	240	R0	CBF
3	F	7	Diaphysis	Ewing	T2 M0 N0	No	No	ISG/AIEOP EW-1	300	R0	CBF
4	F	10	Metaphysis prox	Ewing	T2 M1 N0	Yes	Yes	ISG/AIEOP EW-1	540	R0	VFG
5	F	8	Metaphysis dist	Ewing	T2 M0 N0	No	No	ISG/AIEOP EW-1	280	R0	CBF

Abbreviations: F, female; VFG, vascular fibula graft; CBF, cancellous bone graft; prox, proximal; dist, distal; Mets, metastases; TNM, Tumour, Node, Metastasis.

**Table 2:** Results.

Patient	Follow-Up	Age	Autologous Bone Sparing	Prosthetic Surface	MSTS	Delta Limb Length (cm)	Final Outcome
1	68	14	Proximal humerus epiphysis	Distal humerus osteoarticular	93%	0.8	NED
2	30	17	Diaphysis and distal humerus	Entire shoulder	66%	0	NED
3	27	7	Distal humerus	Proximal humerus osteoarticular	73%	4	NED
4	22	10	Proximal epiphysis and distal humerus metaphysis	Native proximal	83%	2	AWD
				and distal articular surfaces			
5	14	8	Proximal humerus metaphysis	Distal humerus osteoarticular	93%	3	DOD

## Data Availability

The data are not publicly available due to privacy reasons.

## References

[1] Dome J.S., Rodriguez-Galindo C., Spunt S.L., Santana V.M., Niederhuber J.E., Armitage J.O., Kastan M.B., Doroshow J.H., Tepper J.E. (2020). 92—Pediatric Solid Tumors. Abeloff’s Clinical Oncology.

[2] Abed R., Grimer R. (2010). Surgical modalities in the treatment of bone sarcoma in children. Cancer Treat. Rev..

[3] Zhao X., Wu Q., Gong X., Liu J., Ma Y. (2021). Osteosarcoma: A review of current and future therapeutic approaches. BioMed. Eng. OnLine.

[4] Dürr H.R., Bakhshai Y., Rechl H., Tunn P.U. (2014). Resection margins in bone tumors: What is adequate?. Unfallchirurg.

[5] Ogink P.T., Teunissen F.R., Massier J.R., Raskin K.A., Schwab J.H., Lozano-Calderon S.A. (2019). Allograft reconstruction of the humerus: Complications and revision surgery. J. Surg. Oncol..

[6] Barbier D., De Billy B., Gicquel P., Bourelle S., Journeau P. (2017). Is the clavicula pro humero technique of value for reconstruction after resection of the proximal humerus in children?. Clin. Orthop. Relat. Res..

[7] Innocenti M., Delcroix L., Manfrini M., Ceruso M., Capanna R. (2004). Vascularized proximal fibular epiphyseal transfer for distal radial reconstruction. J. Bone Jt. Surg. Am..

[8] Bus M.P., van de Sande M.A., Taminiau A.H., Dijkstra P.D. (2017). Is there still a role for osteoarticular allograft reconstruction in musculoskeletal tumour surgery? A long-term follow-up study of 38 patients and systematic review of the literature. Bone Jt. J..

[9] Hopyan S. (2021). Reconstruction for bone tumours of the shoulder and humerus in children and adolescents. J. Child. Orthop..

[10] Tsuda Y., Fujiwara T., Stevenson J.D., Parry M.C., Tillman R., Abudu A. (2020). The long-term results of extendable endoprostheses of the humerus in children after the resection of a bone sarcoma. Bone Jt. J..

[11] Wafa H., Reddy K., Grimer R., Abudu A., Jeys L., Carter S., Tillman R. (2015). Does total humeral endoprosthetic replacement provide reliable reconstruction with preservation of a useful extremity?. Clin. Orthop. Relat. Res..

[12] Sanchez-Sotelo J., Wagner E.R., Sim F.H., Houdek M.T. (2017). Allograft-prosthetic composite reconstruction for massive proximal humeral bone loss in reverse shoulder arthroplasty. J. Bone Jt. Surg. Am..

[13] Beltrami G., Ristori G., Nucci A.M., Galeotti A., Tamburini A., Scoccianti G., Campanacci D., Innocenti M., Capanna R. (2021). Custom-made 3D-printed implants as novel approach to reconstructive surgery after oncologic resection in pediatric patients. J. Clin. Med..

[14] Brierley J.D., Gospodarowicz M.K., Wittekind C. (2016). TNM Classification of Malignant Tumours.

[15] Henderson E.R., Groundland J.S., Pala E., Dennis J.A., Wooten R., Cheong D., Windhager R., Kotz R.I., Mercuri M., Funovics P.T. (2011). Failure mode classification for tumor endoprostheses: Retrospective review of five institutions and a literature review. J. Bone Jt. Surg. Am..

[16] Enneking W.F., Dunham W., Gebhardt M.C., Malawar M., Pritchard D.J. (1993). A system for the functional evaluation of reconstructive procedures after surgical treatment of tumors of the musculoskeletal system. Clin. Orthop. Relat. Res..

[17] Gautam D., Arora N., Gupta S., George J., Malhotra R. (2021). Megaprosthesis Versus Allograft Prosthesis Composite for the Management of Massive Skeletal Defects: A Meta-Analysis of Comparative Studies. Curr. Rev. Musculoskelet. Med..

[18] Gautam D., Malhotra R. (2018). Megaprosthesis versus allograft prosthesis composite for massive skeletal defects. J. Clin. Orthop. Trauma.

[19] Wang J., Shen J., Dickinson I.C. (2011). Functional outcome of arthrodesis with a vascularized fibular graft and a rotational latissimus dorsi flap after proximal humerus sarcoma resection. Ann. Surg. Oncol..

[20] Innocenti M., Ceruso M., Manfrini M., Angeloni R., Lauri G., Capanna R., Bufalini C. (1998). Free vascularized growth-plate transfer after bone tumor resection in children. J. Reconstr. Microsurg..

[21] Stevenson J.D., Doxey R., Abudu A., Parry M., Evans S., Peart F., Jeys L. (2018). Vascularized fibular epiphyseal transfer for proximal humeral reconstruction in children with a primary sarcoma of bone. Bone Jt. J..

[22] Puri A., Gulia A., Agarwal M., Jambhekar N., Laskar S. (2010). Extracorporeal irradiated tumor bone: A reconstruction option in diaphyseal Ewing’s sarcomas. Indian J. Orthop..

[23] Gupta S., Kafchinski L.A., Gundle K.R., Saidi K., Griffin A.M., Wunder J.S., Ferguson P.C. (2017). Intercalary allograft augmented with intramedullary cement and plate fixation is a reliable solution after resection of a diaphyseal tumour. Bone Jt. J..

[24] Pazourek L., Tomáš T., Mahdal M., Janíček P., Černý J., Ondrůšek Š. (2018). Use of Solid Intercalary Allografts for Reconstruction Following the Resection of Primary Bone Tumors. Acta Chir. Orthop. Traumatol. Cechoslov..

[25] Goulding K.A., Schwartz A., Hattrup S.J., Randall R.L., Lee D., Rispoli D.M., Lerman D.M., Beauchamp C. (2017). Use of compressive osseointegration endoprostheses for massive bone loss from tumor and failed arthroplasty: A viable option in the upper extremity. Clin. Orthop. Relat. Res..

[26] Henrichs M.P., Liem D., Gosheger G., Streitbuerger A., Nottrott M., Andreou D., Hardes J. (2019). Megaprosthetic replacement of the distal humerus: Still a challenge in limb salvage. J. Shoulder Elbow Surg..

[27] Beltrami G. (2018). Custom 3D-printed finger proximal phalanx as salvage of limb function after aggressive recurrence of giant cell tumour. BMJ Case Rep..

[28] Beltrami G., Ristori G., Scoccianti G., Tamburini A., Capanna R., Campanacci D., Innocenti M. (2018). Latissimus dorsi rotational flap combined with a custom-made scapular prosthesis after oncological surgical resection: A report of two patients. BMC Cancer.

[29] Fan H., Fu J., Li X., Pei Y., Li X., Pei G., Guo Z. (2015). Implantation of customized 3-D printed titanium prosthesis in limb salvage surgery: A case series and review of the literature. World J. Surg. Oncol..

[30] Beltrami G., Ristori G., Galeotti A., Scoccianti G., Tamburini A., Campanacci D., Capanna R., Innocenti M. (2021). A hollow, custom-made prosthesis combined with a vascularized flap and bone graft for skeletal reconstruction after bone tumour resection. Surg. Oncol..

[31] Beltrami G., Nucci A.M., Tamburini A., Innocenti M. (2021). Custom-made 3D-printed prosthesis and free vascularised fibula for humeral reconstruction after osteosarcoma resection in a 13-year-old patient. BMJ Case Rep..

